# Evolutionary patterns of two major reproduction candidate genes (*Zp2 *and *Zp3*) reveal no contribution to reproductive isolation between bovine species

**DOI:** 10.1186/1471-2148-11-24

**Published:** 2011-01-25

**Authors:** Shanyuan Chen, Vânia Costa, Albano Beja-Pereira

**Affiliations:** 1 Centro de Investigação em Biodiversidade e Recursos Genéticos da Universidade do Porto (CIBIO/UP), Campus Agrário de Vairão, 4485-661 Vairão, Portugal

## Abstract

**Background:**

It has been established that mammalian egg zona pellucida (ZP) glycoproteins are responsible for species-restricted binding of sperm to unfertilized eggs, inducing the sperm acrosome reaction, and preventing polyspermy. In mammals, ZP apparently represents a barrier to heterospecific fertilization and thus probably contributes to reproductive isolation between species. The evolutionary relationships between some members of the tribe Bovini are complex and highly debatable, particularly, those involving *Bos *and *Bison *species for which interspecific hybridization is extensively documented. Because reproductive isolation is known to be a major precursor of species divergence, testing evolutionary patterns of ZP glycoproteins may shed some light into the speciation process of these species. To this end, we have examined intraspecific and interspecific genetic variation of two ZP genes (*Zp2 *and *Zp3*) for seven representative species (111 individuals) from the Bovini tribe, including five species from *Bos *and *Bison*, and two species each from genera *Bubalus *and *Syncerus*.

**Results:**

A pattern of low levels of intraspecific polymorphism and interspecific divergence was detected for the two sequenced fragments each for *Zp2 *and *Zp3*. At intraspecific level, none of neutrality tests detected deviations from neutral equilibrium expectations for the two genes. Several haplotypes in both genes were shared by multiple species from *Bos *and *Bison*.

**Conclusions:**

Here we argue that neither ancestral polymorphism nor introgressive hybridization alone can fully account for haplotype sharing among species from *Bos *and *Bison*, and that both scenarios have contributed to such a pattern of haplotype sharing observed here. Additionally, codon-based tests revealed strong evidence for purifying selection in the *Zp3 *coding haplotype sequences and weak evidence for purifying selection in the *Zp2 *coding haplotype sequences. Contrary to a general genetic pattern that genes or genomic regions contributing to reproductive isolation between species often evolve rapidly and show little or no gene flow between species, these results demonstrate that, particularly, those sequenced exons of the *Zp2 *and the *Zp3 *did not show any contribution to reproductive isolation between the bovine species studied here.

## Background

It is widely recognized that mammalian egg zona pellucida (ZP) plays critical roles during oogenesis, fertilization, and preimplantation development [1-3]. Initially, three glycoproteins (ZP1-3) were identified and characterized in the mouse egg ZP [4], but recently an additional ZP glycoprotein (ZP4) was also discovered in several mammalian species, such as the laboratory rat, human and primate species [5,6]. Over past two decades, extensive experimental studies have been conducted to assess the role of individual ZP glycoproteins in mediating gamete recognition during fertilization. For instance, ZP3 and ZP2 have been reported as primary and secondary sperm receptors in mice [7]. Some studies further identified a region of mouse ZP3 that possesses sperm receptor activity and defined this region as the 'sperm combing-site' located in the polypeptide encoded by mouse *Zp3 *exon-7 [8-10]. However, some other studies highlight a three-dimensional ZP structure necessary for sperm binding, rather than individual proteins [11-13]. A recent study reveals that sperm-egg recognition in mice depends on the cleavage status of ZP2, but is unaffected by the ZP3 mutations at the sperm combining-site (i.e., the *Zp3 *exon-7) [14].

Although the relative contribution of individual ZP glycoproteins remains arguable, a large body of evidence has established that mammalian ZP glycoproteins are responsible for species-restricted binding of sperm to unfertilized eggs, inducing the sperm acrosome reaction, and preventing polyspermy [8,9]. There is no doubt that mammalian egg ZP represents a barrier to heterospecific fertilization [1], and thus probably somehow contributes to reproductive isolation between species.

It is well known that proteins involved in reproduction, such as those mediating sperm-egg interactions, often have undergone adaptive evolution, driven by positive natural selection [15-18]. Among those ZP glycoproteins in mammals, evidence for rapid evolution resulted from positive selection has been found for both ZP2 and ZP3 [19-22]. In addition, several amino acid sites from both ZPs were identified to be under positive selection [19-22]. Most strikingly, some of positively selected sites in ZP3 are adjacent to or within the sperm combing-site that is essential for binding activity. These results provide support to several nonexclusive hypotheses including sperm competition, cryptic female choice, sexual conflict, reinforcement, avoidance of heterospecific fertilization, and pathogen resistance, to interpret this widespread phenomenon of rapid evolution in reproductive proteins [15,18,23].

In mammals, earlier studies on molecular evolution of reproductive proteins primarily concentrated on diverged species [e.g., [16,21,24]], while more recent studies have shifted to examine genetic variation of reproductive proteins at both intraspecific and interspecific levels [e.g., [25-27]]. Such intra- and inter-specific studies would provide important insights into not just how genetic information flows from generation to generation within species, but also how it has flowed from ancestral species (i.e., the most recent common ancestor) to descendants. Strikingly, there is so far an apparent lack of studies on molecular evolution of reproductive proteins in a group of closely related mammalian species, except for small rodents [19,22,27]. Only few single nucleotide polymorphisms from reproduction-related loci have been reported in several mountain ungulate species [28,29].

Herein we report genetic variation and evolutionary patterns of two egg ZP glycoproteins (ZP2 and ZP3) at both intra- and inter-specific levels in seven representative large mammalian species from the Bovini tribe. The members of this tribe include large animals of great economic and cultural significance to humans such as domestic cattle (*Bos taurus *and *Bos indicus*), Asiatic water buffalo (*Bubalus bubalis*), and the yak (*Bos grunniens*), as well as some of the larger grazers including African buffalo (*Syncerus caffer*), European bison or wisent (*Bison bonasus*), and American bison (*Bison bison*). Despite their importance, the naming of these species from the Bovini tribe is still debatable and for that reason we adopted the classification system suggested by Wilson and Reeder's taxonomic reference [30], complemented by the names of wild versus domestic animals [31]. Indeed, speciation between species from *Bos *and *Bison *is considered incomplete as interspecific hybridization is still occurring [32-36]. The degree of fertility of hybrids or crosses between *Bos *and *Bison *species varies depending on which species pairs are crossed. For instance, both female and male hybrids between *B. taurus *and *B. indicus *are completely fertile, whereas for interspecific hybridization between other *Bos *and *Bison *species female hybrids are fertile and male hybrids are sterile. Thus, these bovine species apparently represent a good model system to test whether ZP glycoproteins contribute to reproductive isolation between such large mammalian species.

## Methods

### Samples and Genomic DNA Extraction

We analyzed a panel of bovine samples including: domestic cattle (*B. taurus *and *B. indicus*), domestic yak (*B. grunniens*), gayal (*B. frontalis*, supposed to be a domestic form of gaur), European bison or wisent (*B. bonasus*), Asiatic water buffalo (*B. bubalis*), and African buffalo (*S. caffer*). Tissue samples were collected from countries across three continents: Yemen (*B. taurus*, n = 9), Turkmenistan (*B. taurus*, n = 4; *B. indicus*, n = 2), Kyrgyzstan (*B. taurus*, n = 5), Kazakhstan (*B. taurus*, n = 5), Mongolia (*B. taurus*, n = 3; *B. indicus*, n = 1), China (*B. grunniens*, n = 11; *B. bubalis*, n = 4), India (*B. indicus*, n = 31; *B. bubalis*, n = 1), Myanmar (*B. frontalis*, n = 6), Egypt (*B. taurus*, n = 3), Morocco (*B. taurus*, n = 15), Kenya (*S. caffer *= 9), and Portugal (*B. bonasus *= 2). Moreover, two individuals each of domestic goat (*Capra hircus*) and sheep (*Ovis aries*) were also included as outgroups. An endeavor was made to avoid sampling related individuals. Genomic DNA was extracted by DNeasy Blood & Tissue Kit (QIAGEN GmbH, Hilden, Germany).

### PCR Amplification and Sequencing

The primer pair Zp2-X8F (5'-CCA TCT CTA CAT GGT GCC TCT-3') and Zp2-X9R (5'-TTG TTT TGA GGA GAG TTT TGC T-3') was used to amplify a fragment spanning the exons 8-9 of *Zp2 *gene, whereas the primer pair Zp3A-X3F (5'-TGC CAT TCA GGA CCA CAG T-3') and Zp3A-X4R (5'-GGA AGT CCA CGA TGG TGT G-3') was used to amplify a fragment spanning the exons 3-4 of *Zp3 *gene. Both primer pairs were designed and tested to be polymorphic across several closely related wild goat species [28]. PCR reactions were performed in a 20 μl volume containing 10X PCR Buffer, 3 (for the *Zp2 *primers) and 1.5 (for the *Zp3 *primers) mM MgCl_2_, 0.2 mM dNTPs, 1 μM each primer, 0.4 U Platinum^® ^*Taq *DNA Polymerase (Invitrogen), and approximately 30 ng genomic DNA. The PCR mixture underwent 10 min at 94 °C, 35 cycles of 30 s at 94 °C, 30 s at 64 °C, and 30 s at 72 °C, and final 10 min at 72 °C on GeneAmp PCR System 9700 (Applied Biosystems, Foster City, CA, USA). PCR products were purified and sequenced for both strands, at the High-Throughput Genomics Unit (HTGU), Department of Genome Sciences, University of Washington (http://www.htseq.org/). Sequence trace files were checked and aligned using software package DNASTAR v7.1 (DNASTAR Inc., Madison, WI, USA). All sequences generated for this study have been deposited in GenBank with accession numbers HM631669-HM631711.

### Data Analyses

Haplotype phases from sequences with heterozygous sites were reconstructed by DnaSP v5.1 [37], which exactly implements a coalescent-based Bayesian method by PHASE 2.1.1 [38,39]. Five independent runs with default options were initially performed to check for consistency between runs and an additional run, with 10 times longer iterations, was conducted to obtain final results. All diversity measures and neutrality tests such as Tajima's *D *[40], Fu and Li's *D *and *F *[41], and Fay and Wu's *H *[42] were calculated at species level, using DnaSP v5.1 [37]. The significance for these neutrality tests was obtained using 10,000 coalescent simulations in DnaSP. The average pairwise differences among seven bovine species for the *Zp2 *and the *Zp3 *sequence data were calculated by Arlequin v3.11 [43].

Phylogenetic relations among haplotype sequences (including non-coding and coding) of the *Zp2 *and the *Zp3 *were independently reconstructed by Bayesian approach as implemented in MrBayes 3.1.2 [44]. The prior best-fitting nucleotide substitution models were separately selected for the coding and non-coding parts of the *Zp2 *and the *Zp3*, respectively. Each model was selected by Modeltest 3.7 [45] based on likelihood ratio tests. The Kimura-2-paramter (K80) model (nst = 2, rates = equal) with different 'TRatio' values was selected for the coding and non-coding parts of the *Zp2*; The K80 model with different 'TRatio' values and 'Rates' were selected for the coding (nst = 2, rates = gamma) and non-coding (nst = 2, rates = equal) parts of the *Zp3*. We applied the selected models with commend 'unlink TRatio = (all) Statefreq = (all)' to the coding and non-coding partitions for both the *Zp2 *and *Zp3 *in MrBayes analyses. Two independent analyses starting from different random trees were performed, and four MCMC chains were run for six million generations with sampling every 100 generations. Six thousand trees were discarded as burn-in, after checking for convergence. Moreover, for the *Zp2 *and the *Zp3 *coding haplotype sequences, we also constructed median-joining networks [46] with program NETWORK version 4.510 (http://www.fluxus-engineering.com/) to display relations among haplotypes.

In addition, we calculated the number of synonymous substitutions per synonymous site (*d*_S_) and the number of nonsynonymous substitutions per nonsynonymous site (*d*_N_) and their variances using bootstrap method with 1000 replications in MEGA4.1 [47,48], for sequence pairs of the *Zp2 *and the *Zp3 *coding haplotypes. With this information, we tested the null hypothesis of neutrality (*d*_N _*= d*_S_) versus alternative hypotheses of positive selection (*d*_N _*> d*_S_) or purifying selection (*d*_N _*< d*_S_) using a Z-test [47,48]. To further detect the presence and the location of selection, the codon-based sequence alignments of the *Zp2 *and the *Zp3 *coding haplotypes were subjected to the sitewise likelihood-ratio (SLR) test [49], guided by a neighbor-joining tree constructed under *p*-distance model in MEGA4.1 [48].

## Results

### Sequence Polymorphism and Divergence

For the *Zp2 *gene, a 310-bp sequence fragment was obtained after trimming the primer sequences, including 64 bp of the exon 8, 88 bp of the intron 8, and 158 bp of the exon 9. There were 26 single nucleotide polymorphisms (SNPs) (11 in non-coding region, 15 in coding region) detected among all sequences including the outgroup species, defining 11 haplotypes (see Additional file 1). Without the outgroup species, there was a total of 15 SNPs (7 in coding region) in seven bovine species.

For the *Zp3 *gene, a 279-bp sequence fragment was obtained after trimming the primer sequences, including 43 bp of the exon 3, 81 bp of the intron 3, and 155 bp of the exon 4. There were 37 SNPs (18 in noncoding region and 19 in coding region) identified among all sequences including the outgroup species, defining 20 haplotype sequences (see Additional file 2). After removal of the outgroup species, there was a total of 25 SNPs (12 in coding region) in seven bovine species. Notably, a two-codon repeat (or a 6-bp insertion of CACACT) in the exon 4 was detected in one individual of taurine cattle from Morocco (African lineage of *B. taurus*).

Low levels of intraspecific polymorphism were found in seven bovine species for the *Zp2 *and the *Zp3 *sequence fragments (Table 1). For example, no any single SNP was detected in 18 chromosomes of African buffalo for the *Zp2 *and in 22 chromosomes of the yak for the *Zp3*, respectively. Moreover, there was also no any single SNP in four chromosomes of the European bison for the two genes. At species level, four neutrality tests based on the frequency spectrum were used to test for deviations from neutral equilibrium expectations. Despite the fact that different neutrality tests focus on different aspects of the frequency spectrum (e.g., Tajima's *D *on rare mutations, Fay and Wu's *H *on high frequency mutations), but we found that none of the tests was significant for the *Zp2 *or the *Zp3 *sequence data across all species, where tests were applicable (Table 1).

**Table 1:** Diversity measures and neutrality tests for the *Zp2 *and the *Zp3 *sequence data*

Gene	Species	*N*	*h*	*Hd*	*S*	π (%)	**θ**_**w **_**(%)**	Tajima's *D*	Fu and Li's *D*	Fu and Li's *F*	Fay and Wu's *H*
***Zp2***	*Bos taurus*	88	2	0.391	2	0.252	0.128	1.532	0.694	1.111	-1.426
	*Bos indicus*	68	3	0.465	4	0.375	0.269	0.835	0.982	1.101	-1.346
	*Bos frontalis*	12	4	0.773	5	0.635	0.534	0.699	1.227	1.168	0.515
	*Bos grunniens*	22	3	0.558	3	0.222	0.265	-0.423	-1.427	-1.344	0.329
	*Bison bonasus*	4	1	0	0	0	0	N.A.	N.A.	N.A.	N.A.
	*Bubalus bubalis*	10	2	0.356	1	0.115	0.114	0.015	0.739	0.667	0.267
	*Syncerus caffer*	18	1	0	0	0	0	N.A.	N.A.	N.A.	N.A.
	*Ovis aries*	2	1	0	0	0	0	N.A.	N.A.	N.A.	N.A.
	*Capra hircus*	2	1	0	0	0	0	N.A.	N.A.	N.A.	N.A.
***Zp3***	*Bos taurus*	88	3	0.365	2	0.134	0.142	-0.088	0.694	0.532	-0.907
	*Bos indicus*	68	7	0.554	7	0.284	0.524	-1.146	0.459	-0.816	0.627
	*Bos frontalis*	12	4	0.712	4	0.600	0.476	0.908	1.227	1.353	0.788
	*Bos grunniens*	22	1	0	0	0	0	N.A.	N.A.	N.A.	N.A.
	*Bison bonasus*	4	1	0	0	0	0	N.A.	N.A.	N.A.	N.A.
	*Bubalus bubalis*	10	5	0.756	5	0.592	0.636	-0.279	-0.527	-0.710	0.889
	*Syncerus caffer*	18	2	0.111	1	0.040	0.105	-1.165	-1.562	-1.690	0.105
	*Ovis aries*	2	1	0	0	0	0	N.A.	N.A.	N.A.	N.A.
	*Capra hircus*	2	1	0	0	0	0	N.A.	N.A.	N.A.	N.A.

* *N *= number of chromosomes; *h *= number of haplotypes; *Hd *= haplotype (gene) diversity; *S *= number of segregating sites; π = nucleotide diversity (per site); θ_w _= Watterson's estimator (per site); N.A. = not applicable. All *P-*values for Tajima's *D*, Fu and Li's *D *and *F *are > 0.10; All *P-*values for Fay and Wu's *H *are > 0.05.

As expected, we also observed low levels of sequence divergence among seven bovine species (Table 2). Among five species from *Bos *and *Bison*, the average pairwise differences ranged from 0.033 to 1.395 for the *Zp2 *and from 0.100 to 4.000 for the *Zp3*. Most distinctly, several haplotypes were shared by multiple species from *Bos *and *Bison*.

**Table 2:** Corrected average pairwise differences among seven species from the Bovini tribe for the *Zp2 *(above diagonal) and the *Zp3 *(below diagonal) sequence data

	1	2	3	4	5	6	7
**1 *Bos taurus***		0.033	1.276	1.108	1.087	4.109	8.087
**2 *Bos indicus***	0.287		1.395	1.243	1.227	4.250	8.227
**3 *Bos frontalis***	0.186	0.100		0.807	0.682	3.704	7.098
**4 *Bos grunniens***	3.633	2.899	2.515		0.156	3.178	7.156
**5 *Bison bonasus***	3.633	2.899	2.848	4.000		3.022	7.000
**6 *Bubalus bubalis***	8.410	7.847	7.743	8.378	8.778		6.022
**7 *Syncerus caffer***	11.042	11.369	10.515	11.000	11.000	8.778	

### Phylogenetic Analyses

**Figure 1:**
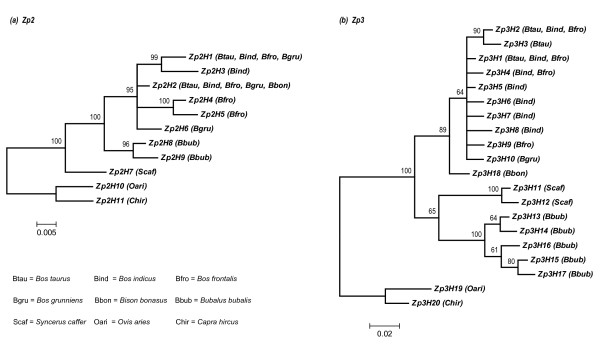
**The Bayesian trees of the *Zp2 *(*a*) and the *Zp3 *(*b*) haplotype sequences from seven species of the Bovini tribe**. The haplotype sequences include both coding and non-coding parts. The domestic sheep and goat are used as the outgroup species. Numbers above nodes indicate posterior probability values.

The Bayesian trees were constructed to display phylogenetic relations among the haplotype sequences (including non-coding and coding) for the *Zp2 *and the *Zp3 *genes (Figure 1). The branching pattern was nearly identical between the two phylogenies. Most noticeably, the haplotype sequences from the same species were not monophyletic. As expected, the five species from *Bos *and *Bison *formed a monophyletic clade, but with unresolved internal relationships. The buffalo (*Bubalus *and *Syncerus*) species versus non-buffalo (*Bos *and *Bison*) species formed reciprocally monophyletic groups in the *Zp3 *phylogeny, being similar to the phylogenetic tree by MacEachern et al [50].

### Network Analyses of Coding Haplotypes

**Figure 2:**
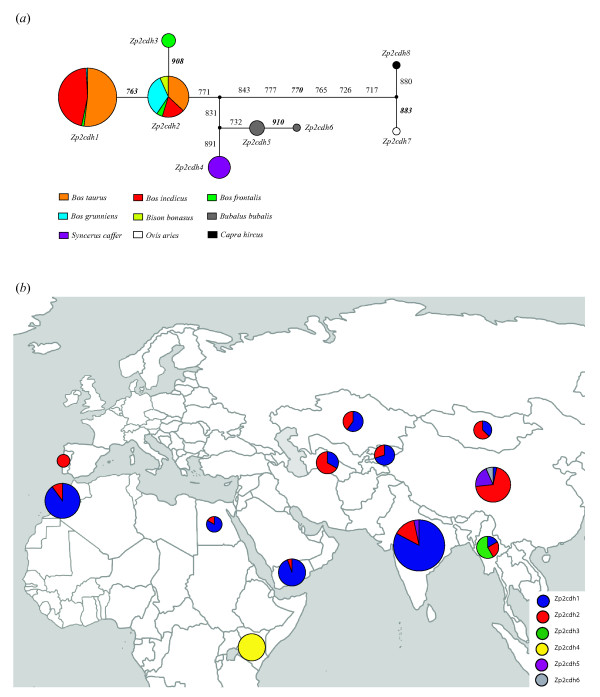
**The median-joining network (*a*) and geographic distribution (*b*) of the *Zp2 *coding haplotypes**. Numbering of mutations follows the full coding sequence of the bovine *Zp2 *gene (from base 1 to 2142). Nonsynonymous mutations are shown in bold and italic. The size of each circle is proportional to its frequency in numbers of chromosomes. The haplotypes *Zp2cdh7 *and *Zp2cdh8 *from the outgroup species are not shown in the geographic map.

**Figure 3:**
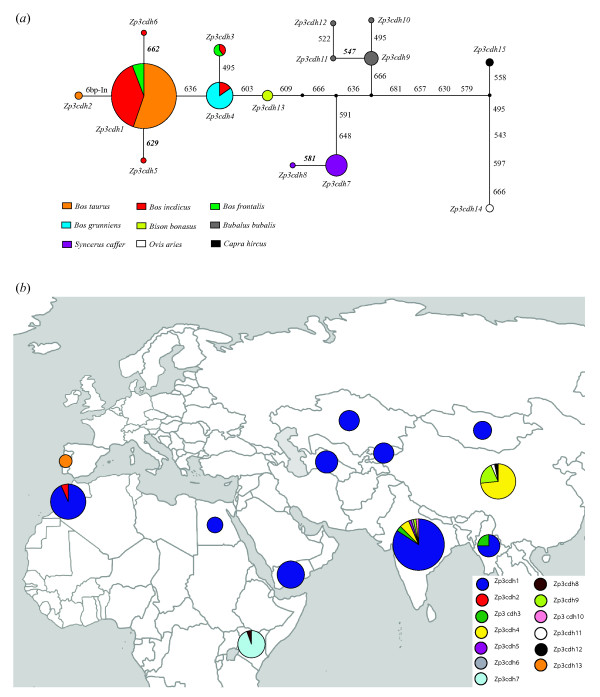
**The median-joining network (*a*) and geographic distribution (*b*) of the *Zp3 *coding haplotypes**. Numbering of mutations follows the full coding sequence of the bovine *Zp3 *gene (from base 1 to 1266). The 6-bp insertion (or two-codon repeat) is located at the relative positions between 609 and 620 or between 603 and 604. Nonsynonymous mutations are shown in bold and italic. The size of each circle is proportional to its frequency in numbers of chromosomes. The haplotypes *Zp3cdh14 *and *Zp3cdh15 *from the outgroup species are not shown in the geographic map.

There were eight coding haplotypes from the 222 bp coding sequence fragment of the *Zp2 *gene, of which the haplotypes *Zp2cdh1 *and *Zp2cdh2 *were predominant and shared by four and five bovine species, respectively (Figure 2a). Noticeably, only three haplotypes were identified in 194 chromosomes of five species from *Bos *and *Bison*. For the *Zp3 *gene, 15 coding haplotypes were defined by 19 coding SNPs (plus the two-codon repeat) in the 198 bp coding sequence fragment, of which one predominant haplotype *Zp3cdh1 *occurred 155 times, shared by *B. taurus*, *B. indicus *and *B. frontalis *(Figure 3a). Interestingly, there was no any single nonsynonymous mutation detected in, at least, 10 mutation steps from the *Zp3cdh1 *to the two outgroup species, i.e., the domestic sheep and goat (Figure 3a).

### Codon-based Tests of Selection

The Z-tests rejected the null hypothesis of neutrality (*d*_N _*= d*_S_) in favor of the alternative hypothesis of purifying selection (*d*_N _*< d*_S_) for the *Zp2 *and the *Zp3 *coding haplotype sequences. Among 28 sequence pairs from the eight *Zp2 *coding haplotypes, 14 were significant at the 5% level for Z-tests, but mainly confined in those sequence pairs with the two outgroup species (see Additional file 3). However, 62 of 91 sequence pairs from the 14 *Zp3 *coding haplotypes were significant at the 5% level for Z-tests, of which 37 were from sequence pairs among seven bovine species (see Additional file 4).

Although evidence for purifying selection has been revealed by Z-tests for both the *Zp2 *and the *Zp3 *coding haplotype sequences, it remains unknown which sites (or codons) have been under purifying selection. We used the SLR method to further calculate the *d*_N_/*d*_S _ratio (i.e., *ω *value) for all codons of the *Zp2 *(74 codons) and the *Zp3 *(66 codons) coding haplotypes. In SLR test, *ω *value <1 indicates purifying selection, while value >1 indicates positive selection [49]. We detected four codons (corresponding to the amino acid positions 176, 179, 185 and 204 of the bovine ZP3 glycoprotein) under purifying selection in the *Zp3 *data, showing *ω *values < 1 with a cutoff >95%, after correcting the SLR test for multiple comparisons. However, no any specific codon was detected to be under purifying selection in the *Zp2 *data.

## Discussion

Our study examined both intra- and inter-specific genetic variation of two sequence fragments each from reproductive genes *Zp2 *and *Zp3*, in seven representative species from the Bovini tribe. A general pattern of low levels of sequence polymorphism and divergence has been detected for those two sequenced fragments (Tables 1 and 2). Due to small sample size for some species studied (e.g., 2 individuals for European bison), the low level of intraspecific variation could be, at first sight, attributable to the effect of sample size. In fact, such a pattern of low polymorphism and divergence was not really ascribed to the sample size, except for an extreme case of European bison, but to the sequence conservation of those two sequenced fragments. Several observations provided strong support to this scenario. First, the sample sizes for domestic cattle (44 for *B. taurus*, 34 for *B. indicus*) were not such small, but the pattern of low levels of intraspecific variation still remained (Table 1). Second, the codon-based Z-tests revealed evidence for purifying selection in pairwise comparisons of the *Zp2 *and the *Zp3 *coding haplotype sequences (see Additional files 3 and 4), suggesting the evolution of the sequenced fragments of the two genes under functional and structural constraints. Finally, the sequenced fragments of the *Zp2 *(from amino acids 238 to 311) and the *Zp3 *(from amino acids 164 to 229) are located at zona pellucida domains, which have been demonstrated to be well conserved in a wide variety of mammalian species [51,52].

It is worthy of note that several haplotypes were shared by multiple bovine species for both genes, for instance, the haplotype *Zp2H2 *(or *Zp2cdh2*) shared by five species from *Bos *and *Bison *(see Figures 1, 2a, and 3a). There were, at least, eight coding SNPs shared by species from *Bos *and *Bison *for the two genes, segregating from the outgroup species, i.e., the domestic goat and sheep (Figures 2a and 3a). Such a pattern of haplotype sharing can be explained by either introgressive hybridization or retention of ancestral polymorphism [53]. The hypothesis of introgressive hybridization is plausible in that interspecific hybridization among species from *Bos *or *Bison *often occurs [32-36]. However, a recent study attempting to reconstruct phylogeny of the Bovini tribe revealed that shared haplotypes among bovine species might be the result of the retention of ancestral polymorphism [50].

To test whether retention of ancestral polymorphism contributed to haplotype sharing among species from *Bos *and *Bison *and to identify specific shared polymorphisms, we looked into patterns of mutations at both sequenced fragments of the *Zp2 *and the *Zp3 *genes. A fixed coding mutation (G > A) at position 5463 of the *Zp2 *was segregated between non-buffalo (*Bos *and *Bison *with 'A') species and buffalo (*Bubalus *and *Syncerus *with 'G') species or the outgroup species (Goat and sheep with 'G') (see Additional file 1); A fixed non-coding mutation (G > A) at position 3270 and a fixed coding mutation (C > T) at 3418 of the *Zp3 *were also completely differentiated between non-buffalo (*Bos *and *Bison *with 'A' at 3270 and 'T' at 3418) species and buffalo (*Bubalus *and *Syncerus *with 'G' at 3270 and 'C' at 3418) species or the outgroup species (Goat and sheep with 'G' at 3270 and 'C' at 3418) (see Additional file 2). It is clear that those three shared polymorphisms among *Bos *and *Bison *species were derived from their most recent common ancestor after divergence from buffalo (*Bubalus *and *Syncerus*) species, but were retained during divergence and evolution of *Bos *and *Bison *species. Thus, such shared polymorphisms provide unequivocal evidence that haplotype sharing among *Bos *and *Bison *species can be, at least, partially attributed to retention of ancestral polymorphism.

In reality, it is difficult to determine whether these shared haplotypes are remnants of ancestral polymorphism or traces of recent introgression events [54], but a recent study suggests that geographic distribution of shared haplotypes would help to disentangle introgression from ancestral polymorphism [55]. To further define a specific shared haplotype due to one of two hypotheses, we mapped all coding haplotypes of both genes on their geographic distribution (Figures 2b and 3b). For the *Zp2*, the shared haplotypes *Zp2cdh1 *(equal to *Zp2H1*, *Zp2H3 *in Figure 1a) and *Zp2cdh2 *(equal to *Zp2H2*, *Zp2H6 *in Figure 1a) are found in samples of multiple *Bos *species across Africa (Morocco and Egypt), Middle East (Yemen), Central Asia (Turkmenistan, Kyrgyzstan, and Kazakhstan), South Asia (India and Myanmar), and Northeast Asia (Mongolia and China). For the *Zp3*, the shared haplotype *Zp3cdh1 *(equal to *Zp3H1*, *Zp3H2*, and *Zp3H9 *in Figure 1b) is also found in samples of multiple *Bos *species across Africa, Middle East, Central Asia, South Asia, and Northeast Asia. However, the shared haplotype *Zp3cdh3 *(equal to *Zp3H4 *in Figure 1b) is only detected in *B. indicus *from India and *B. frontalis *from Myanmar, while the shared haplotype *Zp3cdh4 *(equal to *Zp3H5*, *Zp3H8*, and *Zp3H10 *in Figure 1*b*) is only present in *B. indicus *from India and *B. grunniens *from China. Given that, interspecific hybridization between *Bos *species mainly occurs in South Asia and Central Asia [32-35], and therefore the shared haplotypes *Zp3cdh3 *and *Zp3cdh4 *can be simply explained by hybridization events. Since there is no clear evidence for hybridization between *Bos *species and European bison (*B. bonasus*), although well-documented evidence for hybridization between *B. taurus *and American bison (*B. bison*) exists [36], the haplotype *Zp2cdh2 *shared by *Bos *and *Bison *species is most probably due to retention of ancestral polymorphism, rather than to introgressive hybridization. When scrutinizing phylogenetic relations among coding haplotypes of both genes as shown in networks (see Figures 2a and 3a), both the shared haplotypes *Zp2cdh1 *and *Zp3cdh1 *are located at external nodes of network, compared to the outgroup species through buffalo (*Bubalus *and *Syncerus*) species. Based on the assumption that on average the external haplotypes in the haplotype networks should be younger than those internal ones [54], the haplotypes *Zp2cdh1 *and *Zp3cdh1 *shared by multiple *Bos *species represent relatively recent derived haplotypes at high-frequency, being compatible with a scenario of recent hybridization. Indeed, the recent worldwide spread (less than 10,000 years) of domesticated *Bos *species (and their haplotypes) mediated by humans may have potentiated this scenario. Taken together, none of the two hypotheses alone can fully interpret the presence of shared sequences in the *Zp2 *and *Zp3 *genes among *Bos *and *Bison *species. Indeed, these two hypotheses are reciprocally non-exclusive and therefore both hypotheses can account for our observations of haplotype sharing in the *Zp2 *and *Zp3 *genes among *Bos *and *Bison *species. Our observations of haplotype sharing suggest that adaptive alleles can flow into domestic cattle gene pools from other bovine species via introgressive hybridization, and highlight South Asia as the diversity hotspot for bovine species.

A general genetic pattern of rapid evolution driven by positive nature selection has been revealed for reproductive proteins [15,17,18]. This pattern has also been detected in the ZP2 and the ZP3 glycoproteins of some mammalian species [19-22]. In contrast, we found evidence for purifying (or negative) selection in the sequenced fragments of the *Zp2 *(from amino acids 238 to 311) and the *Zp3 *(from amino acids 164 to 229) genes, rather than positive selection. The molecular signatures of negative selection detected in this study can be easily explained by functional and structural constraints acting on the two sequenced fragments each of the two genes, both of which are located at the conserved ZP domains [51,52].

It should be aware that previous evidence of positive selection in ZP glycoproteins of mammalian species is not robust and consistent among different studies. For instance, a reanalysis of *Mus Zp3 *data from Jansa et al. [19] showed no evidence for positive selection after removal of the outgroup sequences [22]. Berlin and Smith [56] analyzed a *Zp3 *data set of 15 mammalian species including those eight species analyzed by Swanson et al. [21] and found no evidence for positive selection in *Zp3 *gene of those 15 species. In addition, simulation analysis by Berlin and Smith [56] showed that the likelihood ratio test (LRT) comparing M7-M8 that was used by Swanson et al. [21] suffers false positives under fairly simple scenarios, suggesting that the M7-M8 LRT alone does not provide solid evidence for positive selection. It should also be noticed that the M0-M3 LRT used by Swanson et al. [21] is no longer recommended to test evidence of positive selection [57]. To further test whether evidence of positive selection in mammalian *Zp3 *and *Zp2 *genes is robust and consistent, we applied the CODEML site models in PAML version 4.4 [57] to two data sets each for mammalian *Zp3 *(15 species) and *Zp2 *(10 species) full coding sequences, collected from GenBank database (see Additional file 5). Both data sets of *Zp3 *and *Zp2 *genes include those eight mammalian species analyzed by Swanson et al. [21]. Most strikingly, we also found no evidence of positive selection in the *Zp3 *data of 15 species, by the three LRTs comparing M1a-M2a, M7-M8, and M8a-M8 models, respectively (see Additional file 6), being in line with previous results by Berlin and Smith [56]. When considering only those eight species analyzed by Swanson et al. [21], both the M1a-M2a and M8a-M8 LRTs were not significant, whereas the M7-M8 LRT was significant (2ΔlnL(M7-M8) = 7.194, p = 0.027). However, for the *Zp2 *data of 10 species, the M7-M8 and M8a-M8 LRTs were significant (2ΔlnL(M7-M8) = 9.474, p = 0.009; 2ΔlnL(M8a-M8) = 5.030, p = 0.025), but the M1a-M2a LRT was non-significant (2ΔlnL(M1a-M2a) = 3.432, p = 0.180). Likewise, when focusing on only those eight species analyzed by Swanson et al. [21], three LTRs yielded similar results as those from the *Zp2 *data of 10 species (see Additional file 6). Moreover, we identified one site (at 174) in the *Zp2 *data set of 10 species and four sites (at 38, 117, 174, and 342) in the *Zp2 *data of eight species under positive selection with posterior probabilities > 90%. Our analyses on real data sets of both mammalian *Zp3 *and *Zp2 *genes are congruent with previous simulation analyses showing that the M1a-M2a and M8a-M8 comparisons are more robust than the M7-M8 comparison [56,58]. Taken these observations into account, we conclude that there is no evidence of positive selection in *Zp3 *gene of these mammalian species studied, and that there is some (not strong) evidence of positive selection in *Zp2 *gene of these mammalian species studied. It is therefore not strange to find evidence of purifying selection in the sequenced fragment of *Zp3 *gene in bovine species.

We also noticed that evidence for positive selection was detected in *Zp3 *gene of New Guinean and Australasian murine species, by using three LRTs comparing M1a-M2a, M7-M8, and M8a-M8 models [20]. In addition, three amino acid sites (at 324, 325, and 341) within the polypeptide (residues 309-355) encoded by mouse *Zp3 *exon-7 were identified to be under positive selection. The polypeptide encoded by mouse *Zp3 *exon-7 containing sperm combing-site of ZP3 has been demonstrated to be essential for sperm-binding activity by *in vitro *functional experiments [8-10]. However, when considering only Australasian murine species, three LRTs were not significant at all [20]. These results from murine rodents suggest that there is lack of a general pattern of rapid evolution driven by positive selection in *Zp3 *gene across mammalian species. Contrary to mammalian ZP3, we found evidence of positive selection in mammalian ZP2 with several positively selected amino acid sites (at 38, 117, 174, and 342), indicating that mammalian ZP2 might have played a more important role in sperm-egg interaction than previously thought. Although, some authors still support sperm-binding model mediated by ZP3 [2,3,59], such model has been called into questions and is gradually replaced by a three-dimensional ZP structure model [11-13]. The latter model is further supported by a recent study showing that sperm-egg recognition in mouse depends on the cleavage status of ZP2, but is unaffected by the ZP3 mutations in the sperm combining-site [14].

## Conclusions

Previous studies have demonstrated that genes or genomic regions (such as speciation genes) that contribute to reproductive isolation between species often evolve rapidly and show little or no gene flow between species [60,61]. If this postulate is true, then our findings suggest that these two ZP genes may not play an important role in reproductive isolation between the bovine species as it was defended in a recent functional study [14]. Nonetheless, future experiments comparing sperm-binding capacity between bovine species pairs with different degrees of fertility of hybrid offspring are needed. Alternatively, our findings can also be interpreted as the exception to the fast-evolving patterns that were revealed from genes related to reproductive isolation in previous studies. If the latter is true, then our data would appeal a re-evaluation of the ZP evolutionary rates in other mammalian lineages.

## Authors' contributions

SC participated in the design of the study, performed the statistical analysis, drafted and revised the manuscript. VC carried out the laboratory work. AB-P conceived the study, participated in its design and coordination and helped to draft the manuscript. All authors read and approved the final manuscript.

## Supplementary Material

Additional file 1 **Sequence variation of 11 haplotypes of the *Zp2 *310-bp sequence fragment**. The haplotype frequency for each species is listed in the right columns. Numbering of nucleotide sites follows the reference sequence of the *Zp2 *gene from base 1 to 11804 of NC_007326 (i.e., from base 20178291 to 20190094 on *Bos taurus *chromosome 25 [based on Btau_4.0]). The gray-shaded sites are located in coding regions. The abbreviations for species as follows: BTAU = *Bos taurus *(taurine cattle), BIND = *Bos indicus *(zebu cattle), BFRO = *Bos frontalis *(gayal), BGRU = *Bos grunniens *(yak), SCAF = *Syncerus caffer *(African buffalo), BBUB = *Babalus bubalis *(Water buffalo), BBON = *Bison bonasus *(wisent), OARI = *Ovis aries *(sheep), and CHIR = *Capra hircus *(goat). Click here for file

Additional file 2 **Sequence variation of 20 haplotypes of the *Zp3 *279-bp sequence fragment**. The haplotype frequency in each species is listed in the right columns. Numbering of nucleotide sites follows the reference sequence of the *Zp3 *gene from base 1 to 7732 of NC_007326 (i.e., from base 36581113 to 36588844 [based on Btau_4.0]). The gray-shaded sites are located in coding regions. The abbreviations for species are given in the Additional file 1. Click here for file

Additional file 3 Codon-based Z-test of purifying selection (*d*_N _*< d*_S_) for sequence pairs of the *Zp2 *coding haplotypes Click here for file

Additional file 4 **Codon-based Z-test of purifying selection (*d*_N _*< d*_S_) for sequence pairs of the *Zp3 *coding haplotypes**. Click here for file

Additional file 5 **Supplementary methods on PAML analyses of mammalian *Zp3 *and *Zp2 *sequence data from GenBank database**. Click here for file

Additional file 6 **Likelihood ratio tests of codon-substitution models for mammalian *Zp3 *and *Zp2 *data from GenBank database**. Click here for file
