# Supplementary material for: Evolutionary patterns of two major reproduction candidate genes (Zp2 and Zp3) reveal no contribution to reproductive isolation between bovine species
Source: BMC Evol Biol. 2011 Jan 25;11:24. doi: 10.1186/1471-2148-11-24 (PMC3037879; doi:10.1186/1471-2148-11-24)
Supplement: Additional file 1 — Sequence variation of 11 haplotypes of the Zp2 310-bp sequence fragment. The haplotype frequency for each species is listed in the right columns. Numbering of nucleotide sites follows the reference sequence of the Zp2 gene from base 1 to 11804 of NC_007326 (i.e., from base 20178291 to 20190094 on Bos taurus chromosome 25 [based on Btau_4.0]). The gray-shaded sites are located in coding regions. The abbreviations for species as follows: BTAU = Bos taurus (taurine cattle), BIND = Bos indicus (zebu cattle), BFRO = Bos frontalis (gayal), BGRU = Bos grunniens (yak), SCAF = Syncerus caffer (African buffalo), BBUB = Babalus bubalis (Water buffalo), BBON = Bison bonasus (wisent), OARI = Ovis aries (sheep), and CHIR = Capra hircus (goat). [file 1471-2148-11-24-S1.PDF]

	Site	TOTAL	BTAU	BIND	BFRO	BGRU	BBON	BBUB	SCAF	OARI	CHIR
	55555555555555555555555555<br>44444444444455555556666666<br>01255667788900012351266789<br>98457235636356728071303180										
Zp2H11	CGTATTGCTGCATAACACCGCCCCTG	2	0	0	0	0	0	0	0	0	2
Zp2H10	..........T...T......TT...	2	0	0	0	0	0	0	0	2	0
Zp2H7	TA..CC.....GC.T...TATT.T..	18	0	0	0	0	0	0	18	0	0
Zp2H8	TAC.CC..G...CCT...TATT....	8	0	0	0	0	0	8	0	0	0
Zp2H9	TAC.CC..G...CCT...TATT...A	2	0	0	0	0	0	2	0	0	0
Zp2H1	TA.CCCA.G...CCTT..T.TT....	116	65	48	2	1	0	0	0	0	0
Zp2H2	TA..CCA.G...CCT...T.TT....	53	23	11	3	12	4	0	0	0	0
Zp2H3	TA.CCCATGA..CCT...T.TT....	9	0	9	0	0	0	0	0	0	0
Zp2H4	TA..CCA.A...CCT...T.TT..C.	5	0	0	5	0	0	0	0	0	0
Zp2H5	TA..CCA.A...CCT..TT.TT..C.	2	0	0	2	0	0	0	0	0	0
Zp2H6	TA..CCA.G...CCT.G.T.TT....	9	0	0	0	9	0	0	0	0	0

Additional file 1 – Sequence variation of 11 haplotypes of the *Zp2* 310-bp sequence fragment. The haplotype frequency for each species is listed in the right columns. Numbering of nucleotide sites follows the reference sequence of the *Zp2* gene from base 1 to 11804 of NC_007326 (i.e., from base 20178291 to 20190094 on *Bos taurus* chromosome 25 [based on Btau_4.0]). The gray-shaded sites are located in coding regions. The abbreviations for species as follows: BTAU = *Bos taurus* (taurine cattle), BIND = *Bos indicus* (zebu cattle), BFRO = *Bos frontalis* (gayal), BGRU = *Bos grunniens* (yak), SCAF = *Syncerus caffer* (African buffalo), BBUB = *Babalus bubalis* (Water buffalo), BBON = *Bison bonasus* (wisent), OARI = *Ovis aries* (sheep), and CHIR = *Capra hircus* (goat).
