# Supplementary material for: Evolutionary patterns of two major reproduction candidate genes (Zp2 and Zp3) reveal no contribution to reproductive isolation between bovine species
Source: BMC Evol Biol. 2011 Jan 25;11:24. doi: 10.1186/1471-2148-11-24 (PMC3037879; doi:10.1186/1471-2148-11-24)
Supplement: Additional file 3 — Codon-based Z-test of purifying selection (dN < dS) for sequence pairs of the Zp2 coding haplotypes [file 1471-2148-11-24-S3.PDF]

**Additional file 3 - Codon-based Z-test of purifying selection ($d_N < d_S$) for sequence pairs of the *Zp2* coding haplotypes***

| | 1 | 2 | 3 | 4 | 5 | 6 | 7 | 8 |
|---|---|---|---|---|---|---|---|---|
| 1 *Zp2cdh1* | | -1.018 | -1.457 | 1.554 | 1.584 | 1.371 | 2.510 | 3.002 |
| 2 *Zp2cdh2* | 1.000 | | -1.032 | 1.763 | 1.795 | 1.573 | 2.533 | 3.005 |
| 3 *Zp2cdh3* | 1.000 | 1.000 | | 1.553 | 1.579 | 1.368 | 2.345 | 2.819 |
| 4 *Zp2cdh4* | 0.061 | 0.040 | 0.062 | | 1.380 | 1.152 | 2.570 | 2.934 |
| 5 *Zp2cdh5* | 0.058 | 0.038 | 0.058 | 0.085 | | -1.017 | 2.621 | 3.003 |
| 6 *Zp2cdh6* | 0.086 | 0.059 | 0.087 | 0.126 | 1.000 | | 2.478 | 2.859 |
| 7 *Zp2cdh7* | 0.007 | 0.006 | 0.010 | 0.006 | 0.005 | 0.007 | | 0.697 |
| 8 *Zp2cdh8* | 0.002 | 0.002 | 0.003 | 0.002 | 0.002 | 0.003 | 0.244 | |

***** The probability of rejecting the null hypothesis of strict-neutrality ($d_N = d_S$) in favor of the alternative hypothesis ($d_N < d_S$) (below diagonal) is shown. *P*-Values less than 0.05 are considered significant at the 5% level and are highlighted. The test statistic ($d_S - d_N$) is shown above the diagonal. $d_S$ and $d_N$ are the numbers of synonymous and nonsynonymous substitutions per site, respectively.
