# Supplementary material for: Evolutionary patterns of two major reproduction candidate genes (Zp2 and Zp3) reveal no contribution to reproductive isolation between bovine species
Source: BMC Evol Biol. 2011 Jan 25;11:24. doi: 10.1186/1471-2148-11-24 (PMC3037879; doi:10.1186/1471-2148-11-24)
Supplement: Additional file 4 — Codon-based Z-test of purifying selection (dN < dS) for sequence pairs of the Zp3 coding haplotypes. [file 1471-2148-11-24-S4.PDF]

Additional file 4 - Codon-based Z-test of purifying selection ($d_N < d_S$) for sequence pairs of the *Zp3* coding haplotypes*

	1	2	3	4	5	6	7	8	9	10	11	12	13	14
1 *Zp3cdh1*		1.410	0.951	-1.073	-1.028	2.460	2.300	1.824	2.167	1.614	1.947	1.367	3.290	3.175
2 *Zp3cdh2*	0.081		1.036	1.167	1.156	2.573	2.410	2.309	1.997	2.148	2.422	1.492	3.186	3.554
3 *Zp3cdh3*	0.172	0.151		0.628	0.619	2.299	2.122	1.997	2.309	1.825	2.117	1.008	3.451	3.303
4 *Zp3cdh4*	1.000	0.123	0.266		-1.501	2.295	2.144	1.598	1.969	1.401	1.761	1.123	3.228	3.106
5 *Zp3cdh5*	1.000	0.125	0.269	1.000		2.305	2.152	1.602	1.972	1.402	1.758	1.121	3.150	3.028
6 *Zp3cdh6*	0.008	0.006	0.012	0.012	0.011		-1.019	1.950	2.230	1.790	2.101	1.997	3.670	3.110
7 *Zp3cdh7*	0.012	0.009	0.018	0.017	0.017	1.000		1.763	2.061	1.609	1.928	1.808	3.511	2.930
8 *Zp3cdh8*	0.035	0.011	0.024	0.056	0.056	0.027	0.040		1.036	-0.994	0.670	1.694	3.135	2.688
9 *Zp3cdh9*	0.016	0.024	0.011	0.026	0.025	0.014	0.021	0.151		0.674	1.218	2.017	2.851	2.949
10 *Zp3cdh10*	0.055	0.017	0.035	0.082	0.082	0.038	0.055	1.000	0.251		1.035	1.501	2.978	2.522
11 *Zp3cdh11*	0.027	0.008	0.018	0.040	0.041	0.019	0.028	0.252	0.113	0.151		1.842	3.156	2.722
12 *Zp3cdh12*	0.087	0.069	0.158	0.132	0.132	0.024	0.037	0.046	0.023	0.068	0.034		3.202	3.053
13 *Zp3cdh13*	0.001	0.001	0.000	0.001	0.001	0.000	0.000	0.001	0.003	0.002	0.001	0.001		2.477
14 *Zp3cdh14*	0.001	0.000	0.001	0.001	0.002	0.001	0.002	0.004	0.002	0.006	0.004	0.001	0.007	

***** The probability of rejecting the null hypothesis of strict-neutrality ($d_N = d_S$) in favor of the alternative hypothesis ($d_N < d_S$) (below diagonal) is shown. *P*-Values less than 0.05 are considered significant at the 5% level and are highlighted. The test statistic ($d_S - d_N$) is shown above the diagonal. d_S and d_N are the numbers of synonymous and nonsynonymous substitutions per site, respectively.
