# Supplementary material for: Evolutionary patterns of two major reproduction candidate genes (Zp2 and Zp3) reveal no contribution to reproductive isolation between bovine species
Source: BMC Evol Biol. 2011 Jan 25;11:24. doi: 10.1186/1471-2148-11-24 (PMC3037879; doi:10.1186/1471-2148-11-24)
Supplement: Additional file 5 — Supplementary methods on PAML analyses of mammalian Zp3 and Zp2 sequence data from GenBank database. [file 1471-2148-11-24-S5.PDF]

Additional file 5 – Supplementary methods on PAML analyses of mammalian *Zp3* and *Zp2* sequence data from GenBank database

Sequences Retrieved

The *Zp3* and *Zp2* full mRNA sequences of mammalian species were downloaded from GenBank database. A total of 15 and 10 mammalian species were retrieved for *Zp3* and *Zp2*, respectively. The species with GenBank accession numbers are as follows:

Zp3: *Mus musculus* (NM_011776), *Rattus rattus* (Y10823), *Rattus norvegicus* (NM_053762), *Lagurus lagurus* (AF515621), *Lasiopodomys brandtii* (AF304487), *Homo sapiens* (NM_001110354), *Macaca radiate* (X82639), *Callithrix sp* (S71825), *Canis lupus familiaris* (NM_001003224), *Vulpes vulpes* (AY598032), *Felis catus* (NM_001009330), *Sus scrofa* (NM_213893), *Bos grunniens* (GQ856646), *Bos taurus* (NM_173974), *Oryctolagus cuniculus* (NM_001195720). The underlined species were analyzed by Swanson et al. [1].

Zp2: *Mus musculus* (NM_011775), *Rattus norvegicus* (NM_031150), *Homo sapiens* (NM_003460), *Macaca radiate* (Y10690), *Callithrix jacchus* (Y10767), *Felis catus* (NM_001009875), *Canis lupus familiaris* (NM_001003304), *Vulpes vulpes* (AY598031), *Sus scrofa* (NM_213848), *Bos taurus* (NM_173973). The underlined species were analyzed by Swanson et al. [1].

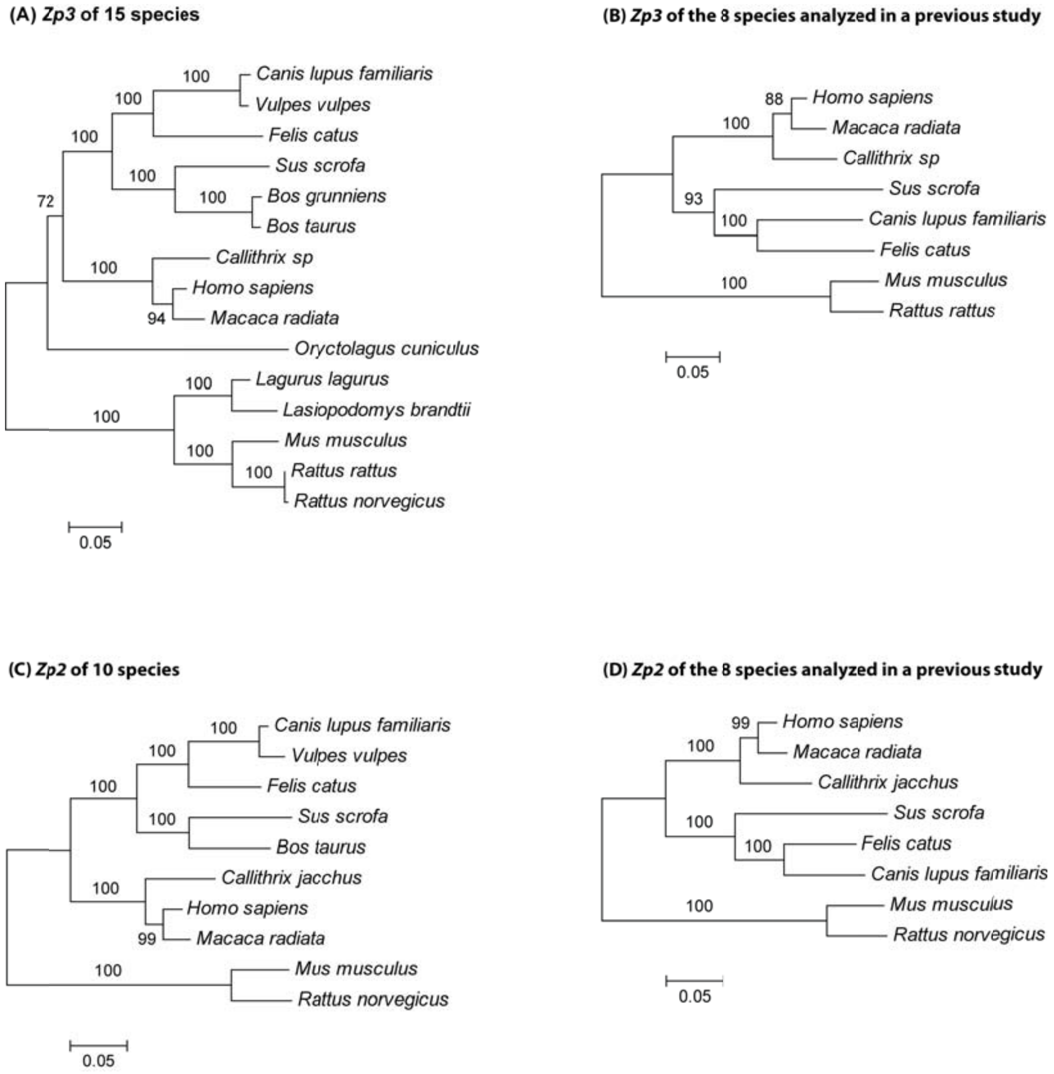

Figure S1 - Maximum likelihood trees of *Zp3* and *Zp2* of mammalian species analyzed in this study. (A) *Zp3* of 15 mammalian species, under TPM2uf+G model ($\alpha = 0.777$); (B) *Zp3* of the 8 species analyzed in a previous study, under TIM2+G model ($\alpha = 0.662$); (C) *Zp2* of 10 species, under TVM+G model ($\alpha = 1.233$); (D) *Zp2* of the 8 species analyzed in a previous study, under TVM+G model ($\alpha = 1.305$). Numbers above nodes indicate bootstrap values with 1000 replications.

Data Analyses

Sequence alignment was initially generated for amino acid sequences by translating full coding sequences (CDSs) into protein sequences, and later translated back to DNA sequences. Amino acid sequences were aligned by PRANKSTER (http://www.ebi.ac.uk/goldman-srv/prank/prankster/) – a graphical interface for the multiple sequence alignment program PRANK [2, 3]. The "+F" option was enforced when making alignment. The program PRANK has been demonstrated to outperform a set of published alignment software programs [3, 4].

Phylogenetic trees were reconstructed by maximum likelihood (ML) method in PAUP*4.0b10 [5]. The best-fit nucleotide substitution models were selected by Akaike information criterion (AIC) with jModelTest version 0.1.1 [6]. ML tree search was heuristic, using 100 random addition analyses with TBR branch-swapping. Bootstrap values for nodes were obtained by 1000 replications using heuristic search and 2 random addition analyses with TBR branch-swapping.

Likelihood ratio tests (LRTs) were conducted to detect evidence for positive selection in mammalian *Zp3* and *Zp2* full coding sequences. The CODEML program in PAML package version 4.4 [7] was implemented to run the site models (M0, M1a, M2a, M7, M8 and M8a). The ML tree topology (Figure S1 as shown above) with branch lengths re-estimated from one-ratio (M0) model was used as user tree for running M1a, M2a, M7, M8 and M8a. The site models allow the ω ratio (i.e., $\omega = d_N/d_S$ – the nonsynonymous/synonymous rate ratio) to vary among codons [8, 9]. The ω ratio measures the direction and strength of selection on amino acid changes, with values of $\omega < 1$, $= 1$, and > 1 indicating purifying (or negative) selection, neutral

evolution, and positive selection, respectively. LRTs compare null models that do not allow for any codons with ω>1 against alternative models that does. Three LRTs (M1a-M2a, M7-M8, and M8a-M8) were carried out to test evidence of positive selection [8-12]. When LRTs show evidence for positive selection, Bayes empirical Bayes (BEB) method can be applied to calculate the posterior probabilities that each codon is from the site class of positive selection under models M2a and M8 [12].

References

1. Swanson WJ, Yang Z, Wolfner MF, Aquadro CF: **Positive Darwinian selection drives the evolution of several female reproductive proteins in mammals**. *Proceedings of the National Academy of Sciences of the United States of America* 2001, **98**(5):2509-2514.
2. Löytynoja A, Goldman N: **An algorithm for progressive multiple alignment of sequences with insertions**. *Proceedings of the National Academy of Sciences of the United States of America* 2005, **102**(30):10557-10562.
3. Löytynoja A, Goldman N: **Phylogeny-Aware Gap Placement Prevents Errors in Sequence Alignment and Evolutionary Analysis**. *Science* 2008, **320**(5883):1632-1635.
4. Fletcher W, Yang Z: **The Effect of Insertions, Deletions, and Alignment Errors on the Branch-Site Test of Positive Selection**. *Molecular Biology and Evolution* 2010, **27**(10):2257-2267.
5. Swofford DL: **PAUP*. Phylogenetic Analysis Using Parsimony (*and Other Methods). Version 4.** Sunderland, Massachusetts: Sinauer Associates; 2002.

6. Posada D: **jModelTest: Phylogenetic Model Averaging**. *Molecular Biology and Evolution* 2008, **25**(7):1253-1256.
7. Yang Z: **PAML 4: Phylogenetic Analysis by Maximum Likelihood**. *Molecular Biology and Evolution* 2007, **24**(8):1586-1591.
8. Nielsen R, Yang Z: **Likelihood Models for Detecting Positively Selected Amino Acid Sites and Applications to the HIV-1 Envelope Gene**. *Genetics* 1998, **148**(3):929-936.
9. Yang Z, Nielsen R, Goldman N, Pedersen A-MK: **Codon-Substitution Models for Heterogeneous Selection Pressure at Amino Acid Sites**. *Genetics* 2000, **155**(1):431-449.
10. Swanson WJ, Nielsen R, Yang Q: **Pervasive Adaptive Evolution in Mammalian Fertilization Proteins**. *Molecular Biology and Evolution* 2003, **20**(1):18-20.
11. Wong WSW, Yang Z, Goldman N, Nielsen R: **Accuracy and Power of Statistical Methods for Detecting Adaptive Evolution in Protein Coding Sequences and for Identifying Positively Selected Sites**. *Genetics* 2004, **168**(2):1041-1051.
12. Yang Z, Wong WSW, Nielsen R: **Bayes Empirical Bayes Inference of Amino Acid Sites Under Positive Selection**. *Molecular Biology and Evolution* 2005, **22**(4):1107-1118.
