# Supplementary material for: Evolutionary patterns of two major reproduction candidate genes (Zp2 and Zp3) reveal no contribution to reproductive isolation between bovine species
Source: BMC Evol Biol. 2011 Jan 25;11:24. doi: 10.1186/1471-2148-11-24 (PMC3037879; doi:10.1186/1471-2148-11-24)
Supplement: Additional file 6 — Likelihood ratio tests of codon-substitution models for mammalian Zp3 and Zp2 data from GenBank database. [file 1471-2148-11-24-S6.PDF]

Additional file 6 - Likelihood ratio tests of codon-substitution models for mammalian *Zp3* and *Zp2* data from GenBank database*

Model	lnL	2ΔlnL M1a-M2a	2ΔlnL M7-M8	2ΔlnL M8a-M8	df	$P(\chi^2)$	Estimates of parameters	Positively selected sites (PP > 90%, PP > 95%*)
**(A) *Zp3* of 15 species**								
M1a	-7722.938						p_0=0.672, p_1=0.328, ω_0=0.100, ω_1=1	Not allowed
M2a	-7722.938	0			2	1.000	p_0=0.672, p_1=0.261, p_2=0.067, ω_0=0.100, ω_1=1, ω_2=1	None
M7	-7706.803						p=0.352, q=0.761	Not allowed
M8	-7704.651		4.304		2	0.116	p_0=0.956, p=0.406, q=1.049, p_1=0.044, ω_s=1.497	None
M8a	-7705.379			1.456	1	0.228	p_0= 0.840, p=0.470, q=1.832, (p_1= 0.160), ω_s=1	Not allowed
**(B) *Zp3* of the eight species analyzed in a previous study**								
M1a	-5600.932						p_0=0.698, p_1=0.302, ω_0=0.096, ω_1=1	Not allowed

M2a	-5600.932	0			2	1.000	p_0=0.698, p_1=0.241, p_2=0.061, ω_0=0.096, ω_1=1, ω_2=1	None
M7	-5603.106						p=0.308, q=0.627	Not allowed
M8	-5599.509		7.194		2	**0.027**	p_0=0.782, p=0.737, q=4.052, p_1=0.218, ω_s=1.102	None
M8a	-5599.612			0.206	1	0.650	p_0=0.742, p=0.853, q=5.593, p_1=0.258, ω_s=1	Not allowed
**(C) *Zp2* of 10 species**								
M1a	-9943.985						p_0=0.550, p_1=0.450, ω_0=0.186, ω_1=1	Not allowed
M2a	-9942.269	3.432			2	0.180	p_0=0.549, p_1=0.437, p_2=0.014, ω_0=0.193, ω_1=1, ω_2=3.041	None
M7	-9945.749						p=0.506, q=0.456	Not allowed
M8	-9941.012		9.474		2	**0.009**	p_0=0.960, p=0.640, q=0.654, p_1=0.040, ω_s=2.260	174S
M8a	-9943.527			5.030	1	**0.025**	p_0=0.598, p=1.572, q=5.069, p_1=0.402, ω_s=1	Not allowed
**(D) *Zp2* of the eight species analyzed in a previous study**								
M1a	-9098.346						p_0=0.550, p_1=0.450,	Not allowed

							ω_0=0.184, ω_1=1	
M2a	-9096.273	4.146			2	0.126	p_0=0.554, p_1=0.427, p_2=0.019, ω_0=0.194, ω_1=1, ω_2=2.845	None
M7	-9101.237						p=0.473, q=0.412	Not allowed
M8	-9095.911		10.652		2	**0.005**	p_0=0.927, p=0.715, q=0.802, p_1=0.073, ω_s=1.950	38P, 117G, 174S, 342G
M8a	-9098.320			4.818	1	**0.028**	p_0=0.561, p=5.138, q=21.031, p_1=0.439, ω_s=1	Not allowed

***** lnL, log likelihood; 2ΔlnL, twice the log likelihood difference between the two compared models; df, degrees of freedom; $P(\chi^2)$, P values of likelihood ratio test (LRT) under χ^2 distribution, significant values (< 0.05); PP, posterior probability, calculated under Bayes Empirical Bayes (BEB) method.
